# Asthma Control in Children Followed in a Tertiary Pediatric Pulmonology Clinic: A Real-World Observational Study

**DOI:** 10.7759/cureus.117356

**Published:** 2026-10-02

**Authors:** Filippos Toliopoulos, Konstantinos Messaritakis, Michail Bizakis, Alexios Giachoudis, Nikolaos Karazafeiris, Stergios Karazafeiris, George Sofianopoulos, Elisavet A Vakouftsi, Kyriaki Papadopoulou-Legbelou, Asterios Kampouras

**Affiliations:** 1 Pediatrics, Aristotle University of Thessaloniki, Thessaloniki, GRC; 2 Pediatrics, University of Thessaly, Larissa, GRC; 3 4th Department of Pediatrics, Aristotle University of Thessaloniki, Thessaloniki, GRC

**Keywords:** asthma, asthma control test, children, pediatric pulmonology, real-world study

## Abstract

Background: Achieving adequate asthma control remains a major challenge in pediatric patients, even among those followed in specialist clinics. Real-world data describing asthma control in routine clinical practice are limited.

Objective: To describe asthma control assessed by the Asthma Control Test (ACT) in children followed in a tertiary pediatric pulmonology clinic and, as exploratory secondary aims, to describe the distribution of age, exacerbation frequency, controller treatment step, and total immunoglobulin E (IgE) in relation to asthma control.

Methods: This retrospective, descriptive observational study included children with physician-diagnosed asthma followed in a tertiary pediatric pulmonology clinic between January 2023 and December 2024. Asthma control was assessed with the standard ACT, administered to all children, and defined as controlled (ACT ≥20) or uncontrolled (ACT <20). Variables included age, number of exacerbations during the previous year, controller treatment step according to the Global Initiative for Asthma (GINA), and total IgE. Continuous variables were summarized as median and interquartile range (IQR). Group comparisons were performed with the Mann-Whitney U and Fisher's exact tests, correlations with Spearman's rank correlation, and 95% confidence intervals (CIs) were reported for all estimates.

Results: A total of 36 children were included; an ACT score was available for 34 (94%), who formed the analysed sample. Twenty children (58.8%) had controlled asthma, and 14 (41.2%, 95% CI 26.4% to 57.8%) had uncontrolled asthma. Children with uncontrolled asthma tended to be older than those with controlled asthma (median age 8.5 vs 6.5 years; median difference 2.0 years, 95% CI -0.5 to 5.0), although this difference did not reach statistical significance (p = 0.052). Step 3 or higher controller therapy was recorded in 8 of 14 children with uncontrolled asthma (57.1%) and 8 of 20 with controlled asthma (40.0%; p = 0.49). In an exploratory analysis, ACT score was inversely correlated with controller treatment step (Spearman ρ = -0.36, 95% CI -0.62 to -0.02; p = 0.04), consistent with treatment escalation in children with more difficult asthma. Exploratory correlation analyses showed a moderate negative correlation between ACT score and total IgE (Spearman ρ = -0.42, 95% CI -0.79 to 0.17; p = 0.15; n = 13) and a weak negative correlation between ACT score and exacerbation frequency (ρ = -0.29, 95% CI -0.57 to 0.05; p = 0.09; n = 34), neither of which reached statistical significance.

Conclusions: A substantial proportion of children followed in a tertiary pediatric pulmonology clinic had suboptimal asthma control despite specialist care. Older children tended to have poorer asthma control, although this difference did not reach statistical significance, and lower ACT scores were associated with higher-intensity controller therapy, most plausibly reflecting treatment escalation in children with more difficult asthma rather than a treatment effect. These exploratory findings highlight the need for age-appropriate asthma management strategies in real-world practice.

## Introduction

Asthma is one of the most common chronic diseases in childhood and remains a major cause of morbidity worldwide [1]. Inadequate asthma control is associated with increased symptom burden, exacerbations, healthcare utilization and impaired quality of life [2]. Achieving and maintaining asthma control is therefore a central goal of pediatric asthma management [2].

In routine clinical practice, asthma control is commonly assessed using validated symptom-based tools such as the Asthma Control Test (ACT), which has been shown to correlate with clinical outcomes and future risk [3,4]. Regular assessment of asthma control is recommended by international guidelines to guide treatment adjustments and follow-up [2].

While randomized controlled trials provide important evidence regarding the efficacy of asthma therapies, real-world data from routine clinical settings are essential to understand treatment outcomes in unselected pediatric populations, particularly in tertiary care settings where disease severity and complexity may be greater [5,6]. Despite specialist follow-up, many children continue to experience suboptimal asthma control.

The primary aim of this descriptive, hypothesis-generating study was to report the proportion of children with controlled and uncontrolled asthma, as assessed by the ACT, in a cohort followed in a tertiary pediatric pulmonology clinic. Secondary, exploratory aims were to describe the distribution of age, exacerbation frequency, controller treatment step and total immunoglobulin E (IgE) in relation to asthma control.

## Materials and methods

Study design and setting

This was a retrospective, descriptive observational study conducted in the Pediatric Pulmonology Clinic of the General Hospital "Papageorgiou", Thessaloniki, Greece, a tertiary referral center for pediatric respiratory disease. Data collection covered a 24-month period, from January 2023 to December 2024.

Study population

Children with a physician diagnosis of asthma who were followed in the pediatric pulmonology clinic during the study period were eligible for inclusion; 36 children met these criteria and were included. A consecutive (convenience) sampling technique was used: all eligible patients seen in the clinic during the study period were considered for inclusion, with no additional randomization or stratification. Patients were excluded if they had an alternative or additional chronic respiratory diagnosis that could confound the assessment of asthma control (e.g., cystic fibrosis, bronchopulmonary dysplasia, primary ciliary dyskinesia), if a physician diagnosis of asthma could not be confirmed from the medical record, or if the ACT could not be reliably administered or interpreted (e.g., because of a developmental or cognitive limitation). An ACT score recorded at the index visit was available for 34 of the 36 children (94%); these 34 children constitute the analysed sample for all analyses involving asthma control. The two children without a recorded ACT score are included only in the description of the overall cohort.

Data collection

Clinical data were extracted from medical records and included age (years), ACT score, number of asthma exacerbations in the previous year, controller treatment step and, where available, total serum IgE. Asthma was diagnosed by the treating pediatric pulmonologist according to the Global Initiative for Asthma (GINA) 2023 report [2]. Controller treatment step was classified according to the GINA stepwise approach (steps 1-5) as documented at the index visit; children not receiving any controller medication were recorded as "no controller therapy". Adherence to controller medication was assessed clinically during clinic visits on the basis of patient and caregiver report and was not measured objectively.

Assessment and definition of asthma control

Asthma control was assessed with the standard five-item ACT [3,4], which was administered to all children as part of routine clinic practice. In children younger than 12 years, the questionnaire was completed by the child together with a parent or caregiver, in accordance with the established clinic routine at the time of the study; the age-specific Childhood Asthma Control Test (cACT) [7] was not used. Both the ACT and the cACT define uncontrolled asthma as a score of 19 or less; asthma was accordingly classified as controlled (ACT ≥20) or uncontrolled (ACT <20). This cut-off is consistent with prior validation studies and guideline recommendations [3,4].

Confounders

Given the retrospective, exploratory design and modest sample size, formal multivariable adjustment for potential confounders was not performed. Controller treatment step, a clinically relevant covariate that may influence both disease severity and asthma control, was recorded and is reported descriptively by group rather than statistically adjusted for. Other potential confounders, such as comorbid allergic rhinitis, environmental tobacco smoke exposure, obesity, socioeconomic status and objectively measured medication adherence, were not systematically captured in the dataset and could not be assessed; this is acknowledged as a limitation in the Discussion.

Statistical analysis

Continuous variables are presented as median and interquartile range (IQR), and categorical variables as numbers and percentages. Comparisons between the controlled and uncontrolled asthma groups were performed with the Mann-Whitney U test for continuous variables and Fisher's exact test for categorical variables. Correlations between clinical and laboratory variables were assessed with Spearman's rank correlation coefficient because of non-normal distributions and the limited sample size. Ninety-five percent confidence intervals (CIs) were calculated with the Wilson method for proportions, by Fisher's z transformation for Spearman correlation coefficients, and by bootstrap resampling (5,000 resamples) for the difference in medians between groups. The rank-biserial correlation is reported as the effect size for the Mann-Whitney U test. In three children, the number of exacerbations was documented as "more than 4" or "more than 5"; because Spearman correlation and the Mann-Whitney U test are rank-based, these values were ranked according to the documented lower bound. All correlation analyses were considered exploratory. A p-value < 0.05 was considered statistically significant. No a priori sample size calculation was performed; as an exploratory, hypothesis-generating study, all eligible consecutive patients seen during the study period were included (convenience sample). All statistical analyses were performed using Python (version 3.11; SciPy and statsmodels libraries).

Ethical approval

This study was approved by the Scientific Council of the General Hospital "Papageorgiou" (approval number ΕΣ 415η/29-07-2026). The requirement for individual informed consent was waived for this retrospective review of anonymized clinical data; informed consent for treatment and open-access publication was obtained or waived for all participants in accordance with institutional policy.

## Results

Cohort characteristics and asthma control

A total of 36 children were included in the study. The median age of the cohort was 8.0 years (IQR 5.0-10.0) and the median number of asthma exacerbations during the previous year was 1.0 (IQR 1.0-2.0). An ACT score was available for 34 children (94%), who formed the analysed sample; the median ACT score was 20.0 (IQR 17.2-23.0). Total IgE was available for 15 children (median 354 IU/mL, IQR 267-913). The characteristics of the cohort overall and by asthma control status are summarized in Table 1.

**Table 1:** Characteristics of the study cohort overall and by asthma control status Values are median (IQR) or n (%); n denotes the number of children with the variable available. Age, exacerbations and IgE: Mann–Whitney U test; treatment step ≥3: Fisher's exact test. Overall values for ACT score and treatment step refer to the 34 children with an ACT score. ACT, Asthma Control Test; IgE, immunoglobulin E; IQR, interquartile range.

Characteristic	Overall cohort (N = 36)	Controlled asthma (ACT ≥20), n = 20	Uncontrolled asthma (ACT <20), n = 14	p value
Age, years	8.0 (5.0–10.0); n = 36	6.5 (4.0–9.0)	8.5 (8.0–9.8)	0.052
ACT score	20.0 (17.2–23.0); n = 34	23.0 (21.0–24.0)	16.0 (14.2–18.0)	—
Exacerbations in previous year, n	1.0 (1.0–2.0); n = 36	1.0 (0.8–2.0)	1.0 (1.0–2.0)	0.58
Total IgE, IU/mL	354 (267–913); n = 15	311 (244–460); n = 4	857 (334–1170); n = 9	0.20
Controller treatment step ≥3, n (%)	16/34 (47.1%); n = 34	8 (40.0%)	8 (57.1%)	0.49

Among the 34 children with an ACT score, 20 (58.8%) had controlled asthma, and 14 (41.2%, 95% CI 26.4-57.8%) had uncontrolled asthma. Children with uncontrolled asthma tended to be older than those with controlled asthma (median 8.5 years, IQR 8.0-9.8, vs 6.5 years, IQR 4.0-9.0; Figure 1). The median difference was 2.0 years (bootstrap 95% CI -0.5 to 5.0; rank-biserial correlation 0.40), but the difference did not reach statistical significance (Mann-Whitney U test, p = 0.052). The distribution of control status by age group is shown in Table 3: uncontrolled asthma was recorded in 1 of 10 children aged 5 years or younger, 11 of 20 children aged 6-11 years, and 2 of 4 children aged 12 years or older. There was no significant difference in exacerbation frequency between the controlled and uncontrolled groups (median 1.0, IQR 0.8-2.0, vs 1.0, IQR 1.0-2.0; p = 0.58).

**Figure 1:**
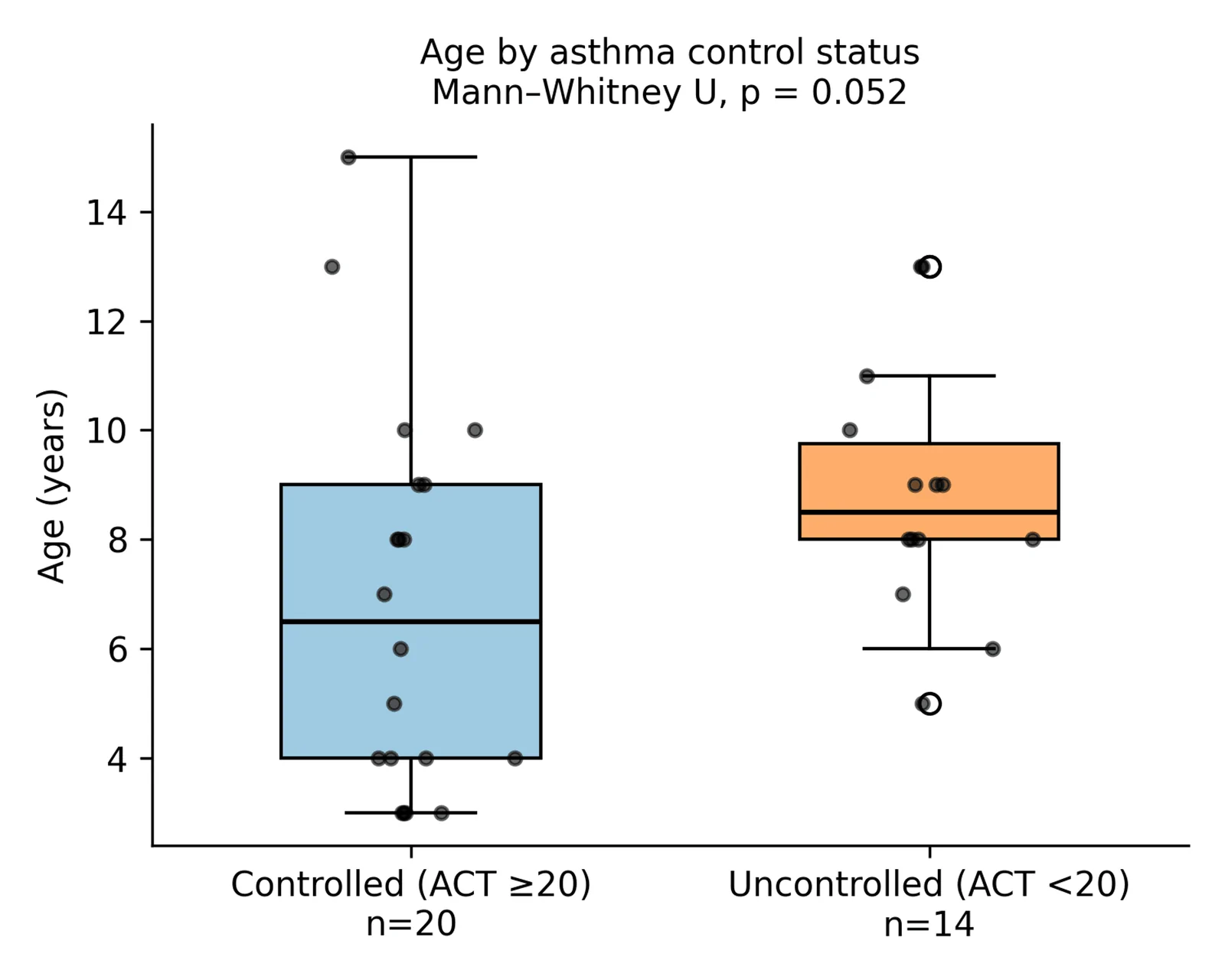
Age distribution by asthma control status Box plots (median, interquartile range, range) with individual data points of age in children with controlled asthma (ACT ≥20, n = 20) and uncontrolled asthma (ACT <20, n = 14). Children with uncontrolled asthma tended to be older (median 8.5 vs 6.5 years; median difference 2.0 years, bootstrap 95% CI -0.5 to 5.0), but the difference did not reach statistical significance (Mann-Whitney U test, p = 0.052). ACT, Asthma Control Test.

Controller treatment step

Controller treatment step was documented for all 34 children in the analysed sample (Table 2). Step 3 or higher controller therapy was recorded in 8 of 14 children with uncontrolled asthma (57.1%) and in 8 of 20 children with controlled asthma (40.0%) (Fisher's exact test, p = 0.49); the median treatment step was 3.0 (IQR 2.0-3.0) in the uncontrolled group and 2.0 (IQR 0.8-3.0) in the controlled group (Mann-Whitney U test, p = 0.17).** **In an exploratory analysis, ACT score was inversely correlated with controller treatment step (Spearman ρ = -0.36, 95% CI -0.62 to -0.02; p = 0.04); this was the only association in the study that reached statistical significance and, as an unadjusted correlation among several exploratory analyses, it should be interpreted with caution.

**Table 2:** Controller treatment step by asthma control status (n = 34) Values are n (%) or median (IQR). Treatment step classified according to GINA 2023 [2] as documented at the index visit. Step ≥3 versus lower: Fisher's exact test, p = 0.49. Median step: Mann–Whitney U test, p = 0.17. ACT, Asthma Control Test; GINA, Global Initiative for Asthma; IQR, interquartile range.

Controller treatment step (GINA)	Controlled asthma (ACT ≥20), n = 20	Uncontrolled asthma (ACT <20), n = 14
No controller therapy	5 (25.0%)	2 (14.3%)
Step 1	4 (20.0%)	1 (7.1%)
Step 2	3 (15.0%)	3 (21.4%)
Step 3	8 (40.0%)	6 (42.9%)
Step 4	0 (0.0%)	2 (14.3%)
Step ≥3, n (%)	8 (40.0%)	8 (57.1%)
Median step (IQR)	2.0 (0.8–3.0)	3.0 (2.0–3.0)

**Table 3:** Asthma control by age group (n = 34) Values are n (%). Uncontrolled asthma defined as ACT <20. ACT, Asthma Control Test. This is an original figure generated by the authors from the study dataset using Python (matplotlib).

Age group	n	Uncontrolled asthma (ACT <20), n (%)
≤5 years	10	1 (10.0%)
6–11 years	20	11 (55.0%)
≥12 years	4	2 (50.0%)
Total	34	14 (41.2%)

Exploratory correlation analyses

In exploratory analyses, a moderate negative correlation was observed between ACT score and total IgE (Spearman ρ = -0.42, 95% CI -0.79 to 0.17; p = 0.15; n = 13; Figure 2), although the very small sample size limits the statistical power and warrants cautious interpretation of this finding. A weak negative correlation was observed between ACT score and the number of asthma exacerbations during the previous year (ρ = -0.29, 95% CI -0.57 to 0.05; p = 0.09; n = 34). Neither correlation reached statistical significance. Aeroallergen sensitization data were available for too few children, and in too heterogeneous a format, to be analysed. 

**Figure 2:**
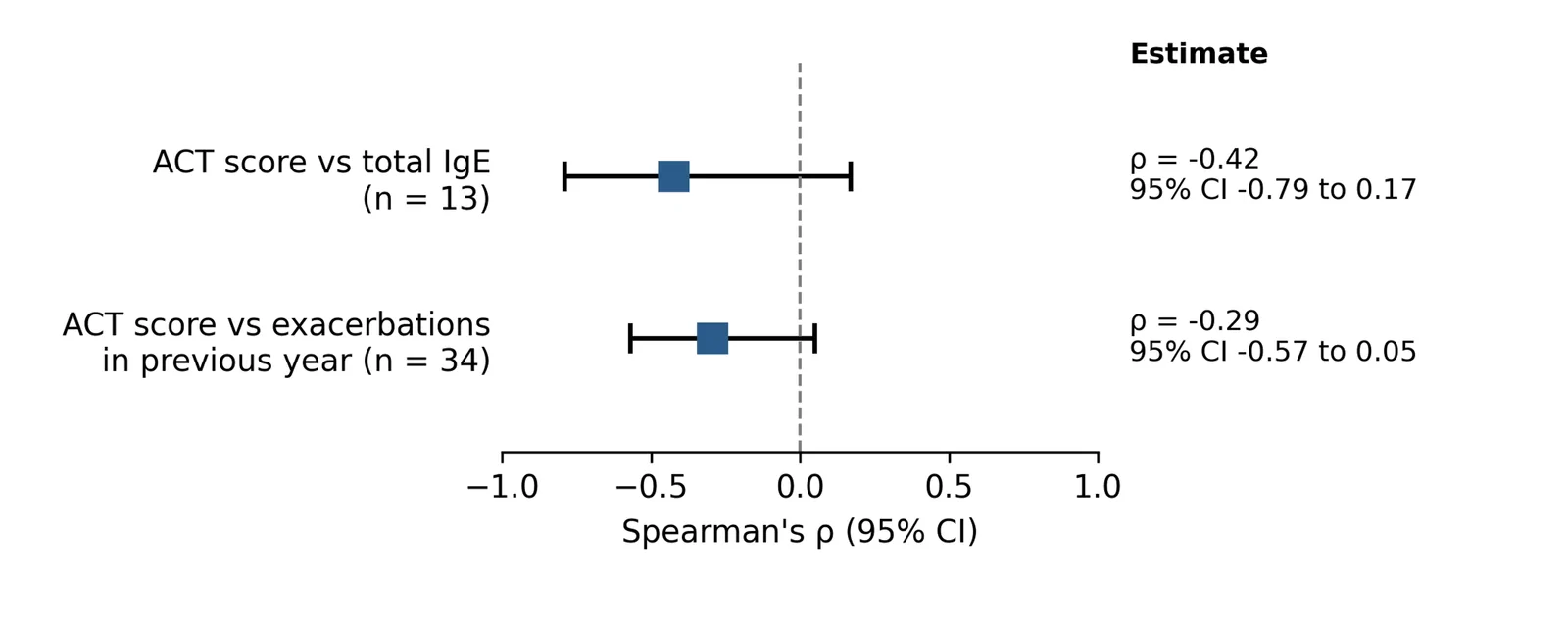
Forest plot of exploratory Spearman correlations with ACT score Spearman correlation coefficients (squares) with 95% confidence intervals (Fisher z transformation) for the correlation of ACT score with total IgE (ρ = -0.42, 95% CI -0.79 to 0.17; n = 13) and with the number of asthma exacerbations in the previous year (ρ = -0.29, 95% CI -0.57 to 0.05; n = 34). Neither correlation reached statistical significance. Analyses are exploratory given the limited number of observations. ACT, Asthma Control Test; CI, confidence interval; IgE, immunoglobulin E. This is an original figure generated by the authors from the study dataset using Python (matplotlib).

## Discussion

In this real-world observational study, more than 40% of children followed in a tertiary pediatric pulmonology clinic had uncontrolled asthma as assessed by the ACT. This finding highlights the persistent burden of suboptimal asthma control even among children receiving specialist care and is consistent with the proportions of uncontrolled asthma reported in real-world surveys of European patients [6].

Older children in our cohort tended to have poorer asthma control, although the difference did not reach statistical significance (Figure 1, Table 3). This observation is consistent with previous studies showing that school-aged children and adolescents may be at increased risk of poor asthma control compared with younger children [8,9], but it requires confirmation in a larger sample. Increased autonomy, psychosocial factors and environmental exposures may contribute to this pattern. In our cohort the pattern was driven mainly by children aged 6-11 years, since only four children were 12 years or older.

Adherence to controller medication is a critical determinant of asthma control and may be particularly challenging in older children and adolescents. Adherence in this study was assessed clinically during clinic visits and relied on patient and caregiver report; it was not measured objectively by electronic monitoring, prescription refill records or drug-level assays. Self-reported adherence is known to overestimate actual medication use [10], and studies using objective monitoring methods have demonstrated lower adherence rates than those reported by patients, particularly in adolescents [11,12]. Reduced parental supervision, concerns regarding medication side effects and social stigma may all contribute to suboptimal adherence in this age group [12]. Consequently, our data cannot distinguish children whose asthma is genuinely resistant to their prescribed treatment step from children in whom poor control reflects unrecognized non-adherence, and no inference about treatment resistance should be drawn from the distribution of treatment steps in Table 2.

The only statistically significant association in this study was the inverse correlation between ACT score and controller treatment step, and the proportion of children receiving Step 3 or higher therapy was somewhat larger in the uncontrolled group. Because controller therapy in routine practice is stepped up in response to persistent symptoms, this finding most plausibly reflects appropriate treatment escalation in children with more difficult asthma, that is, confounding by indication, rather than an effect of treatment on control. It is also an unadjusted correlation from a small sample in which several exploratory associations were examined, so the result is compatible with chance and should not be over-interpreted. Nevertheless, it is clinically relevant in that a substantial fraction of children remained uncontrolled despite receiving medium- or high-intensity therapy, which, together with the reliance on self-reported adherence, highlights the need to verify adherence and inhaler technique before further escalation.

The exploratory correlation analyses did not demonstrate statistically significant associations between ACT score and total IgE or exacerbation frequency (Figure 2). Although a moderate negative correlation between ACT and IgE was observed, the confidence interval was wide and included zero, the estimate was based on 13 children, and the finding should be regarded as hypothesis-generating only. These observations are consistent with the multifactorial nature of asthma control, in which symptom burden and inflammatory biomarkers may not align consistently in real-world practice [5].

This study has several limitations. It was retrospective and single-center, and the sample was small, so that all comparisons and correlations were underpowered and are presented as exploratory. Potential confounders of asthma control, including comorbid allergic rhinitis, environmental tobacco smoke exposure, obesity, socioeconomic factors and objectively measured adherence, were not systematically recorded and could not be adjusted for; residual confounding therefore cannot be excluded. Biomarker data were available for a minority of children, and aeroallergen sensitization data were too sparse and heterogeneously recorded to be analysed. An important further limitation is that the standard ACT was used across the whole age range. The ACT has been validated in patients aged 12 years and older, whereas the cACT is the validated instrument for children aged 4-11 years [7]; the scores of younger children in our cohort, who were assessed with parental assistance, should therefore be interpreted with caution, and this may have influenced the classification of control in this age group. The findings should therefore be regarded as descriptive and hypothesis-generating. Nevertheless, the study provides real-world insight into asthma control among children followed in a Greek tertiary pediatric pulmonology clinic, a setting for which such data have not previously been reported.

## Conclusions

In this tertiary pediatric pulmonology cohort, a substantial proportion of children had suboptimal asthma control despite specialist follow-up. Older children tended to have poorer asthma control, although this difference did not reach statistical significance. Lower ACT scores were associated with higher-intensity controller therapy, an observation that most plausibly reflects treatment escalation in children with more difficult asthma, particularly among older children. Because adherence was assessed by self-report only, these observations cannot distinguish true treatment resistance from unrecognized non-adherence.

An exploratory, non-significant inverse correlation between ACT score and total IgE was observed in the small subgroup with available biomarker data; this observation is hypothesis-generating only. Systematic capture of biomarker and sensitization data in routine clinic workflows, together with age-appropriate assessment of asthma control and objective measurement of adherence, would allow these questions to be addressed adequately in future studies.
